# Acupuncture and massage combined with rehabilitation therapy for hemiplegia after stroke: A protocol for systematic review and meta-analysis

**DOI:** 10.1097/MD.0000000000028732

**Published:** 2022-02-11

**Authors:** Chang Liu, Tingting Pang, Junjie Yao, Jiahui Li, Siyuan Lei, Jiangchun Zhang, Yufeng Wang, Jing Bian

**Affiliations:** a Department of Acupuncture and Tuina, Changchun University of Chinese Medicine, Changchun, China; b Department of Tuina, the Affiliated Hospital to Changchun University of Chinese Medicine, China; c Changchun University of Chinese Medicine, Changchun, China.

**Keywords:** acupuncture, hemiplegia, massage, meta-analysis, rehabilitation, stroke, systematic review

## Abstract

**Background:**

The purpose of this study was to evaluate the effectiveness and safety of acupuncture and massage combined with rehabilitation in the treatment of hemiplegia after stroke.

**Methods:**

To collect relevant literature, we will research following databases: Medicine, PubMed, Embase, Web of Science, Cochrane Library, China National Knowledge Infrastructure, Wan-Fang Database, Chongqing VIP Chinese Science and Technology Periodicaols Database, and China Biomedical Database; the time is from its creation to May 2021, and the language is limited to Chinese and English. In addition, we will retrieve other literature resources, including the Chinese Clinical Trial Register and conference articles. Two reviewers will independently complete the literature screen and data extraction and quality assessment of the included studies will be independently completed by two other researchers. The primary outcomes included the Modified Ashworth scale and the simplified Fugl-Meyer Assessment scale. The Modified Barthel Index, the China Stroke Scale, and adverse reactions as secondary outcomes were assessed. RevMan V.5.4.1 software will be used for meta-analysis, and the Grading of Recommendations Assessment, Development and Evaluation (GRADE) will be used to assess the quality of evidence.

**Results:**

This systematic review will provide a high-quality synthesis to evaluate the efficacy and safety of acupuncture and massage combined with rehabilitation in the treatment of hemiplegia after stroke, providing a reference for the safe and effective treatment of hemiplegia after stroke.

**Conclusion:**

This study provides evidence that acupuncture and massage combined with rehabilitation therapy is effective.

**Ethics and dissemination:**

The protocol of the systematic review does not require ethical approval because it does not involve humans. This article will be published in peer-reviewed journals and presented at relevant conferences.

**Systematic review registration:**

INPLASY202210026.

## 1 Introduction

Stroke is a syndrome of limited or generalized cerebral deficits due to acute cerebral circulatory disorders. It is one of the common cranial lesions with long latency period, rapid onset, high disability rate and high mortality rate,^[1]^ and the incidence of stroke in China is increasing year by year with the accelerated aging of the population and the change of people's lifestyle, of which about 80% are ischemic strokes.^[2]^ Local cerebrovascular lesions in patients with ischemic stroke affect the neurological function of the body, such as hemiplegia,^[3]^ and according to statistics,^[4]^ 50% of patients have reduced mobility due to hemiplegia, which not only seriously affects the quality of life of patients, but also brings a huge burden to families and society.

Presently, there is no specific treatment for stroke hemiplegia, but rehabilitation training is the main treatment, combined with certain drugs, physiotherapy, and surgery to promote functional recovery, but the treatment period is often long and the results are not satisfactory in some patients.^[5]^Studies have pointed out that effective rehabilitation training at an early stage can promote the recovery of neurological function to a certain extent and is beneficial to the recovery of limb function in stroke patients.^[6]^ With the application of acupuncture and massage techniques in patients with ischemic stroke, their effectiveness and safety have been recognized and affirmed.^[7,8]^ In recent years, an increasing number of clinical studies have used integrative therapies to intervene in the disease, with acupuncture and massage combined with modern rehabilitation therapy being a popular choice. Although many clinical studies have reported its positive effects on post-stroke hemiplegia, there is no scientific evidence. Therefore, this systematic review aims to evaluate the effectiveness and safety of acupuncture and massage combined with rehabilitation for post-stroke hemiplegia and to provide a better basis for clinical decision-making.

## 2 Methods and analysis

The study was conducted following the guidelines of the Preferred Reporting Items for Systematic Review and Meta-analysis Protocol (PRISMA-P).^[9]^ This study protocols have been funded through a protocol registry. This protocol of the systematic review has been registered on the INPLASY website. Registration: INPLASY202210026.

### 2.1 Inclusion criteria

#### 2.1.1 Types of participants

All patients should be diagnosed with stroke and show symptoms of hemiplegia, and should be older than 18 years of age. However, race, sex, and educational status are not limited. The diagnosis of stroke should meet WHO criteria.^[10]^ Participants with unstable vital signs or inability to cooperate with rehabilitation treatment should be excluded, such as patients with impaired hearing, visual and cognitive or severe infection, organ dysfunction, and so on.

#### 2.1.2 Types of interventions

The intervention in the experimental group should be acupuncture and massage combined with rehabilitation therapy and interventions of the control group should only be rehabilitation therapy. The methods of rehabilitation training are not limited (including all types of rehabilitation training methods for hemiplegia after stroke, such as Bobath Technology, Rood Technology, Brunnstrom Therapy, Excercise Relearning Therapy and Proprioceptive Neuromuscular Facilitation). If there are other adjuvant therapies, the 2 groups should be consistent.

#### 2.1.3 Types of studies

Inclusion: We will include only randomized controlled clinical trials (RCTs) of Acupuncture and massage combined with rehabilitation therapy for hemiplegia after stroke.

Exclusion: We will exclude any other literature including non-randomized clinical controlled trials, retrospective research literature, conference abstracts, case reports, repeated published literature, and literature of information without data.

#### 2.1.4 Types of outcomes

##### 2.1.4.1 Main outcomes

We will include the Modified Ashworth Scale (MAS) and Simplified Fugl-Meyer Assessment scale (SFMA) as the main outcomes. The MAS will be used to evaluate the muscle tone of the patient's limbs and divided into 5 grades according to severity. The SFMA, 100 points in total, can assess movement function of patient's limbs (including upper and lower limbs).

##### 2.1.4.2 Secondary outcomes

1. Modified Barthel Index used to evaluate the daily living ability of patients with stroke.
2. China Stroke Scale used to assess the neurological deficit of stroke patients.
3. Adverse reactions.

### 2.2 Data sources and search methods

#### 2.2.1 Electronic searches

We will collect relevant articles by searching the following databases: PubMed, Web of Science, Medicine, EMBASE, Cochrane Library, China National Knowledge Infrastructure, China Biomedical Literature Database, China Science Journal Database, and Wan-Fang Database. All databases will be searched from creating to May 1, 2021, by the following words: Stroke, Post-stroke, Apoplexy, Cerebrovascular disorder, Brain ischemia, Intracranial arterial disease, Hemiplegia∗, Monoplegia∗, Flaccid Hemiplegia, Acupuncture, Massage, Tuina, Acupoint, Meridians, Rehabilitation, Habilitation, RCT, and so on. The research strategy for PubMed is presented in Table 1.

**Table 1:** Search strategy used in PubMed.

**No**	Search items
**#1**	Stroke (All Fields)
**#2**	Post-stroke (All Fields)
**#3**	Apoplexy (All Fields)
**#4**	Cerebrovascular disorder (All Fields)
**#5**	Brain ischemia (All Fields)
**#6**	Intracranial arterial disease (All Fields)
**#7**	Intracranial embolism and thrombosis (All Fields)
**#8**	Intracranial haemorrhages (All Fields)
**#9**	#1 OR #2-#8
**#10**	Hemiplegia∗ (All Fields)
**#11**	Monoplegia∗ (All Fields)
**#12**	Flaccid Hemiplegia (All Fields)
**#13**	#10 OR #11 OR #12
**#14**	Acupuncture (All Fields)
**#15**	Massage (All Fields)
**#16**	Tuina (All Fields)
**#17**	Acupoint (All Fields)
**#18**	Meridians (All Fields)
**#19**	Rehabilitation (All Fields)
**#20**	Habilitation (All Fields)
**#21**	#14 OR #15-#20
**#22**	Randomized controlled trial (All Fields)
**#23**	Controlled clinical trial (All Fields)
**#24**	Randomized (All Fields)
**#25**	Randomly (All Fields)
**#26**	#22 OR #23-#25
**#27**	#9 AND #13 AND #21 AND #26

#### 2.2.2 Searching for other resources

We will search the reference list of the included studies and existing systematic reviews related to our topic. We will also search for other literature resources, including the Chinese Clinical Trial Register, conference articles and other related gay literature to make our search as complete as possible.

### 2.3 Data collection and export

**Figure 1:**
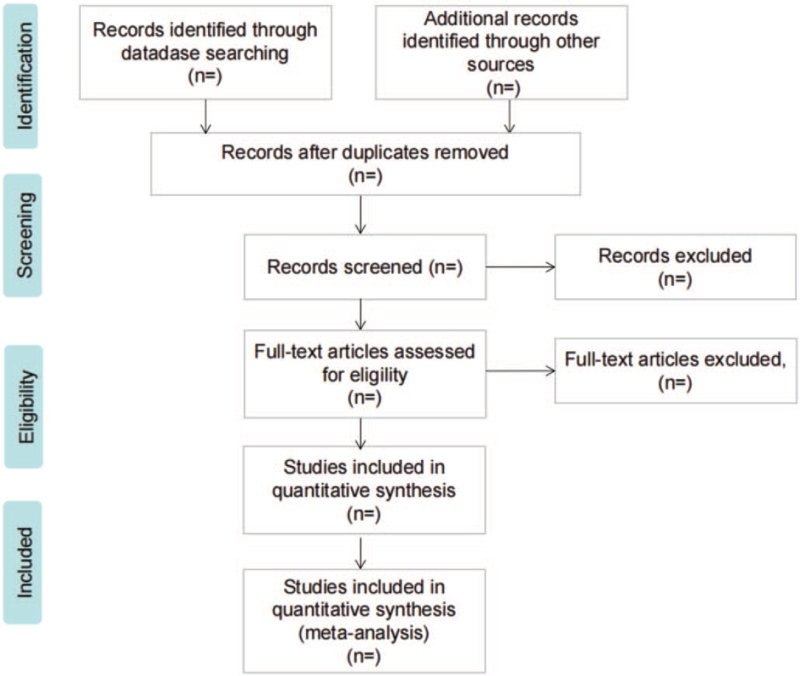
Flow diagram of study selection process.

Two researchers independently screened the literature according to the eligibility criteria. First, they eliminated duplicate articles using EndNote V.x 9.0 and excluded articles that did not meet the inclusion criteria by reading the title and subject. Second, they will perform a screen again of the remaining articles by reading the full text according to the inclusion and exclusion criteria and determine whether it is available for the systematic review. We will also record the excluded papers and explain the reasons for this; the specific screening process is shown in Figure 1. If there is disagreement during, the third researcher will be invited to make a decision.

### 2.4 Data extraction and analysis

Data extraction will be performed by two reviewers independently, and the results will be cross-matched. When the differences and opinions are inconsistent, they should be settled through discussion. If the differences encountered cannot be resolved through discussion, a third researcher will be invited to resolve them. We will make an Excel to extract data which includes the first author, country, year of publication, patient characteristics, number of participants, interventions, outcomes, results, main conclusions, conflicts of interest, ethical approval, and other information. If necessary, we will contact the corresponding author by e-mail to obtain more accurate data.

### 2.5 Assessment of risk of bias in the included studies

Two researchers will independently evaluate the bias risk, including studies using the assessment tool of risk bias in the Cochrane Handbook V.5.1.0. The contents included random sequence generation, allocation sequence concealment, blinding of participants and personnel, outcome assessors, incomplete outcome data, selective outcome reporting, and other sources of bias. The assessment results will be rated as low-risk, high-risk and uncertain risk. In the process, if there is disagreement, a third reviewer will be invited to make a decision.

### 2.6 Assessment of heterogeneity

The heterogeneity test will be carried out among all studies included using the *I*^2^ statistic. When *I*^2^ was <50%, there was no significant heterogeneity. Otherwise, if the result of *I*^2^ is >50%, we believe that there is obvious heterogeneity and subgroup analysis and sensitivity analysis will be conducted to investigate the sources of heterogeneity.

### 2.7 Assessment of reporting biases

We will analyze the quality of publication bias using Rev Man 5.4.1 software in inverted funnel plots and performing Egger test when there were >10 trials included in the meta-analysis.

### 2.8 Data synthesis

The meta-analysis of data from included outcomes will be performed using the RevMan V.5.4.1 and we will choose a randomized or fixed-effect model for data statistics according to the results of the heterogeneity test. The enumeration data were expressed as relative risk, and the weight mean difference was used as the measurement data; each effect amount was expressed in 95% confidence interval. The specific methods were as follows: If the heterogeneity was low (*I*^2^ < 50%, the fixed-effects model was used for data synthesis. If there is high heterogeneity (*I*^2^ > 50%), the random-effects model will be used for data synthesis after excluding possible heterogeneity sources. The investigation methods included subgroup and sensitivity analyses. If data cannot be synthesized, we provide a descriptive analysis to solve this problem.

### 2.9 Subgroup analysis

If there was high heterogeneity (*I*^2^ >50%) among the included studies, we conducted a subgroup analysis to analyze the sources of heterogeneity according to the following factors: age, sex, race, courses, sample sizes, different methods of aromatherapy massage, and other possible factors affecting the results.

### 2.10 Sensitivity analysis

To test the stability and reliability of the results of this study, we conducted a sensitivity analysis according to the following points: method quality, sample size, and missing data. After that, we will perform a data analysis again and compare the results. If there was no directional change after the sensitivity analysis, the results were stable.

### 2.11 Grading the quality of evidence

We will use the Grading of Recommendations Assessment, Development and Evaluation to access confidence in cumulative evidence.^[11]^ The risk of publication, heterogeneity, indirectness, imprecision, and publication bias were assessed and the results were divided into 4 levels: high, moderate, low, and very low.

### 2.12 Ethics and dissemination

Ethical approval will not be required, as no primary information of individual patients was collected. We will publish this article in a peer-reviewed journal.

## 3 Discussion

Stroke is the second most common cause of death and the third most common cause of disability, posing a serious threat to human health.^[12–15]^ Although the death rate of stroke has improved with the present level of treatment, the disability rate is still high. In recent years, the number of stroke disability cases in all age groups has increased,^[16,17]^ with hemiplegia being the main cause of disability in stroke patients.^[18]^ The commonly used treatment method is rehabilitation training, but there are defects such as long treatment period and easy to leave sequelae. Acupuncture and massage, as a non-pharmacological therapy in TCM, has been widely used in clinical practice, and research results have shown^[7,8]^ that acupuncture and massage have better efficacy on post-stroke hemiplegia. The combination of traditional acupuncture and massage with modern rehabilitation techniques can greatly shorten the clinical treatment cycle and improve the therapeutic effect. However, due to the lack of a structured approach, this conclusion still needs to be supported by valid evidence. This study will conduct a systematic review and meta-analysis of data from relevant randomized controlled trials to verify its effectiveness and safety and provide evidence-based medical evidence for clinical treatment of this disease.

## Author contributions

Jing Bian and Yufeng Wang contributed to the conception of this study. Chang Liu drafted and revised the manuscript. The search strategy was developed by all the authors and will be performed by Junjie Yao and Jiahui Li, Siyuan Lei and Jiangchun Zhang will independently screen the potential studies and extract data from the included studies. Assess the risk of bias and complete Tingting Pang. Chang Liu will complete data synthesis. Yufeng Wang arbitrate disagreements. All authors approved the publication of the protocol.

**Data curation:** Siyuan Lei.

**Formal analysis:** Jiangchun Zhang.

**Funding acquisition:** Yufeng Wang and Jing Bian.

**Investigation:** Tingting Pang.

**Methodology:** Junjie Yao and Jiahui Li.

**Validation:** Yufeng Wang and Chang Liu.

**Writing – original draft:** Chang Liu.

**Writing – review & editing:** Jing Bian.
